# Antioxidative, Anti-Inflammatory, and Anti-Aging Properties of Mycosporine-Like Amino Acids: Molecular and Cellular Mechanisms in the Protection of Skin-Aging

**DOI:** 10.3390/md17040222

**Published:** 2019-04-12

**Authors:** Hakuto Kageyama, Rungaroon Waditee-Sirisattha

**Affiliations:** 1 Department of Chemistry, Faculty of Science and Technology, Meijo University, 1-501 Shiogamaguchi, Tenpaku-ku, Nagoya, Aichi 468-8502, Japan; 2 Department of Microbiology, Faculty of Science, Chulalongkorn University, Phayathai Road, Pathumwan, Bangkok 10330, Thailand

**Keywords:** mycosporine-like amino acids, mycosporine-2-glycine, UV-absorbing compound, sunscreen, anti-aging, anti-oxidation, anti-inflammation, anti-protein-glycation activity

## Abstract

Prolonged exposure to ultraviolet (UV) radiation causes photoaging of the skin and induces a number of disorders, including sunburn, fine and coarse wrinkles, and skin cancer risk. Therefore, the application of sunscreen has gained much attention to reduce the harmful effects of UV irradiation on our skin. Recently, there has been a growing demand for the replacement of chemical sunscreens with natural UV-absorbing compounds. Mycosporine-like amino acids (MAAs), promising alternative natural UV-absorbing compounds, are a group of widely distributed, low molecular-weight, water-soluble molecules that can absorb UV radiation and disperse the absorbed energy as heat, without generating reactive oxygen species (ROS). More than 30 MAAs have been characterized, from a variety of organisms. In addition to their UV-absorbing properties, there is substantial evidence that MAAs have the potential to protect against skin aging, including antioxidative activity, anti-inflammatory activity, inhibition of protein-glycation, and inhibition of collagenase activity. This review will provide an overview of MAAs, as potential anti-aging ingredients, beginning with their structure, before moving on to discuss the most recent experimental observations, including the molecular and cellular mechanisms through which MAAs might protect the skin. In particular, we focus on the potential anti-aging activity of mycosporine-2-glycine (M2G).

## 1. Introduction

The skin, the largest human organ, is constantly exposed to the external environment. Exposure to a variety of environmental stress factors, particularly ultraviolet (UV) radiation in sunlight, can damage skin. Sunlight can be broken down into three types of nonionizing electromagnetic radiation—infrared (IR) (780–3000 nm), visible (400–780 nm), and UV (100–400 nm). The percentages of energy radiated to the Earth, in the total energy emitted by the Sun, are 53% IR, 39% visible, and 8% UV [1]. On the basis of its physiological and biological effects, UV radiation can be further divided into three main bands—the 315–400 nm band (designated as UV-A), the 280–315 nm band (designated as UV-B), and the 100–280 nm band (designated as UV-C) [2]. Solar UV radiation is drastically diminished as it passes through the ozone layer and the atmosphere; as a result, the proportion of UV rays in the sunlight reaching the Earth’s surface, is made up of 95% UV-A and 5% UV-B [1]. Although it comprises only a small portion of the total UV radiation, UV-B is thought to be more harmful than UV-A, since UV-B is most active in damaging the skin and eyes [3]. UV-A and UV-B are also known to be genotoxic, meaning they can induce photochemical damage in cellular DNA and proteins [4,5]. Consequently, exposure to UV-A and UV-B, stimulates skin photoaging and can be responsible for the induction of skin cancer [6]. Skin photoaging is characterized by the development of pigmentary disorders, such as solar lentigines, fine and coarse wrinkles, and benign, premalignant, and malignant skin tumors on sun-exposed skin [7]. Highly energetic UV-C radiation has no biological significance, because it does not reach the Earth’s surface, due to its complete absorption by the ozone layer and the atmosphere [1,3]. The depletion of the ozone layer, over the past few decades, has increased the amount of solar UV radiation reaching the Earth’s surface [8], in particular UV-B levels, since UV-A is not absorbed by the ozone layer [9].

Many marine organisms that are exposed to UV radiation have developed photoprotective mechanisms [10]. For example, in cyanobacteria, which dominate the marine environment, UV protection mechanisms have evolved at the molecular, cellular, and behavioral levels [11]. Cyanobacteria can synthesize various types of “sunscreen” compounds, which confer protection against UV radiation. Mycosporine-like amino acids (MAAs), scytonemin, and carotenoids are known to be key compounds in cyanobacteria that can absorb wavelengths in the UV range. These natural products are promising candidate molecules in the field of cosmeceutical compounds discovery [12]. In fact, MAAs have already been commercialized as Helioguard^®^365. This cosmetic reagent contains the liposomal MAAs, shinorine (SHI), and porphyra-334 (P334), that were originally extracted from the red alga *Porphyra umbilicalis*, and has been successfully commercialized as a natural and safe sunscreen compound [12]. Additionally, MAAs are thought to be multifunctional secondary metabolites, in the cells of producers [13]. Many MAAs are known to act as antioxidants [14], while several recent reports have suggested that MAAs have potential therapeutic applications for reducing skin-aging processes. From this point of view, recently, several review reports with special emphasis on the potential use of MAAs in cosmetic products, have been published [15,16,17]. In this paper, we review MAAs and their potential as anti-skin-aging ingredients, by describing a basic overview of their structure, before moving on to a detailed account of the most recent experimental observations, accumulated thus far. The mechanisms by which MAAs might act to protect the skin from aging is discussed at, both, the cellular and the molecular level. In particular, the prominent potential anti-aging activity of the MAA mycosporine-2-glycine (M2G), which is biosynthesized by the halotolerant cyanobacterium *Aphanothece halophytica*, is highlighted.

## 2. Mechanisms of the UV-Induced Skin Aging

UV radiation is important for our health as UV-B exposure can induce the production of a crucial nutrient, vitamin D, in our skin [18]. However, long-term and repeated exposure to UV can promote the skin photoaging process, including skin cancer formation [19]. The mechanisms by which UV-mediated cellular damage is induced are briefly described in this section.

### 2.1. Cellular DNA Damage

Direct and indirect toxic effects of UV radiation on the DNA molecule, mediate photoaging. Direct absorption of UV-B photons by DNA, can result in the generation of pyrimidine dimers, leading to defects in the DNA strand [20]. UV-B radiation leads mostly to the formation of *cis-syn* cyclobutadipyrimidines (CPDs) and pyrimidine (6-4) pyrimidone photoproducts (6-4PPs). 6-4PPs can be converted into related Dewar valence isomers (DewPPs), upon UV-excitation at 314 nm. Such DNA damage interferes with DNA replication and transcription, and brings various harmful effects to the cell, such as mutation, instability of the chromosome, and cell death. UV-A does not directly alter the structure of DNA as DNA does not strongly absorb radiation in the UV-A range [21]. However, UV-A can damage DNA indirectly, via a photosensitized reaction mediated by generating the radical singlet oxygen (^1^O_2_), resulting in purine base modifications [20]. The singlet oxygen anion oxidizes the guanine moiety, followed by the generation of 8-oxo-7,8-dihydroguanine (8-oxo-G) and 8-oxo-7,8-dihydro-2’-deoxyguanosine (8-oxo-dG). As 8-oxo-G and 8-oxo-dG can associate with adenine instead of cytosine, transition mutations might occur.

### 2.2. Reactive Oxygen Species Generation

Reactive oxygen species (ROS), initiators of oxidative stress, are oxygen-containing reactive chemical species that include hydrogen peroxide (H_2_O_2_), hydroxyl radicals (∙OH), superoxide anion radicals (·O_2_^−^), and ^1^O_2_. In our skin, exposure to UV radiation is known to be associated with the generation of ROS. These ROS can activate skin-aging cascades, such as matrix metalloproteinase (MMP)-1-mediated aging and NF-κB-TNF-α-mediated, inflammation-induced aging [22]. The variety of ROS generation mechanism by UV, depend on the UV radiation wavelength range. In addition to ^1^O_2_ generation, as mentioned above, it has been reported that UV-A radiation can induce the generation of ∙O_2_^−^ by the activation of intracellular nicotinamide adenine dinucleotide phosphate (NADPH) oxidase, NOX [23], and association with advanced glycation end-products (AGEs) [24]. Sakurai et al. elucidated that both ^1^O_2_ and ∙O_2_^-^ were generated in the skin of mice exposed to UV-A [25]. H_2_O_2_, ∙O_2_^−^, and ∙OH species might be generated in AGEs, during exposure to UV-A [24]. UV-B is also known to lead to the production of H_2_O_2_, ∙O_2_^−^, and ∙OH [26]. Although the source of these UV-B-induced ROS remains unclear, recently it was reported that NADPH oxidase, NOX1, is associated with UV-B-induced p38/MAPK activation and cytotoxicity, via ROS generation in keratinocytes [26].

**Figure 1:**
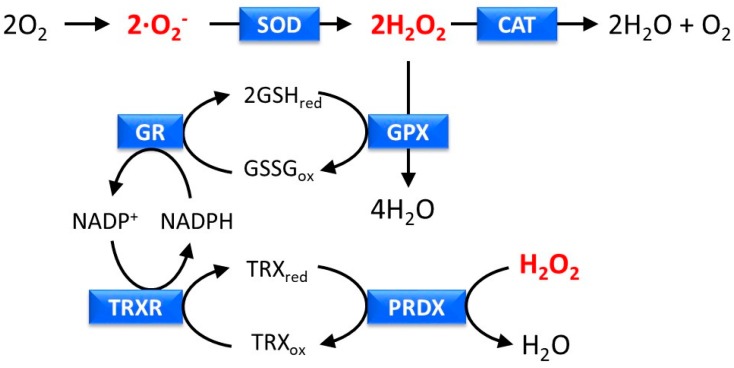
Removal of reactive oxygen species (ROS) by an antioxidant defense system consisting of superoxide dismutase (SOD), catalase (CAT), glutathione peroxidase (GPX), glutathione reductase (GR), thioredoxin oxidase (TRXR), and peroxiredoxin (PRDX). GSHred and GSSGox indicate reduced glutathione and oxidized glutathione, respectively. TRXred and TRXox indicate reduced thioredoxin and oxidized thioredoxin, respectively.

To prevent skin damage induced by excess UV-induced ROS and regulate epidermal homeostasis, skin cells possess an antioxidative function that acts as an endogenous defense system [20]. This system mainly consists of six enzymes—superoxide dismutase (SOD), catalase (CAT), glutathione peroxidase (GPX), glutathione reductase (GR), thioredoxin oxidase (TRXR), and peroxiredoxin (PRDX) (Figure 1). SOD and CAT eliminate ∙O_2_^−^ and H_2_O_2_, respectively, and ultimately convert ∙O_2_^−^ to H_2_O, whereas GPX, GR, TRXR, and PRDX eliminate H_2_O_2_, by regulation of the redox conditions of glutathione and thioredoxin. In addition to this enzymatic system, non-enzymatic molecules, such as vitamin C (ascorbic acid), vitamin E (α-tocopherol), glutathione, and ureic acid, play a major role as antioxidants in the skin [27]. These small molecules scavenge and neutralize free radicals, by providing an extra electron to make an electron pair.

### 2.3. Inflammatory Responses

**Figure 2:**
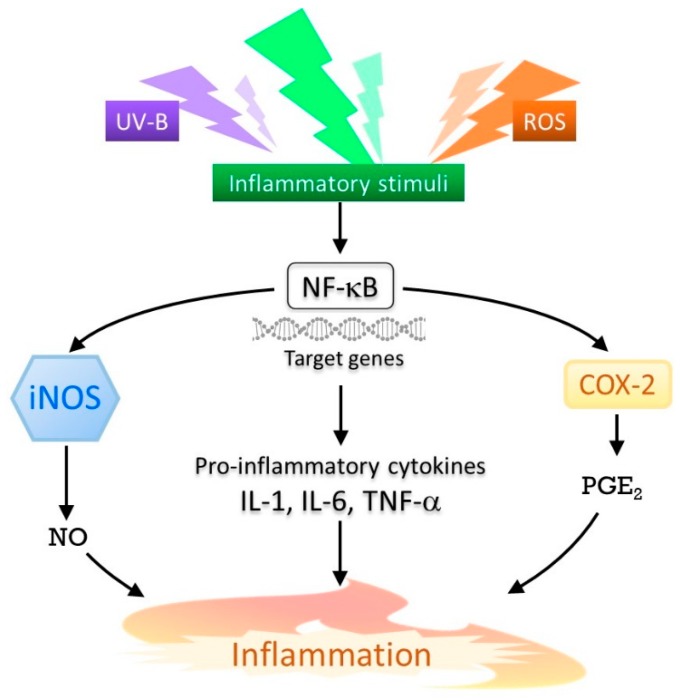
UV-B-induced inflammatory response. Nitric oxide (NO), inducible NO synthase (iNOS), prostaglandin E2 (PGE_2_), cyclooxygenase-2 (COX-2), tumor necrosis factor-α (TNF-α), and other cytokines, such as interleukin-1 (IL-1) and interleukin-6 (IL-6), are shown as mediators.

Exposure to UV radiation induces inflammation by triggering chemical reactions in the skin. Distinct patterns of inflammation are caused by exposure to specific wavelengths of light. The three groups, UV-A, UV-B, and UV-C, have been classified, based on these different patterns of inflammation [28]. Erythema induced in the skin, following exposure to UV-B radiation is characterized as sunburn. Inflammatory responses induced by UV-B are mostly achieved through a variety of mediators, including nitric oxide (NO), inducible NO synthase (iNOS), prostaglandin E2 (PGE_2_), cyclooxygenase-2 (COX-2), tumor necrosis factor-α (TNF-α), and other cytokines, such as interleukin-1 (IL-1) and interleukin-6 (IL-6) (Figure 2). These molecules are predominantly regulated by nuclear factor-kappa B (NF-κB), and mostly produced in keratinocytes, which are the predominant cell type in the epidermis [29]. It has been reported that the expression of the COX-2 protein, which is responsible for PGE_2_ production, is upregulated, following exposure to UV-B, in both human skin and in cultured human keratinocytes [30]. ROS are also known to be associated with the inflammatory response, as it has been observed that COX-2 expression was induced by ROS in different cell types [31].

### 2.4. Induction of Matrix Metalloproteinases 

UV radiation upregulates the expression of matrix metalloproteinases (MMPs) in the skin. MMPs, which are known to be responsible for the destruction of extracellular matrix (ECM) proteins, such as collagen, play an important role in maintaining skin homeostasis and skin aging [32]. MMPs are secreted by keratinocytes and dermal fibroblasts, in response to multiple stimuli, including oxidative stress and cytokines, in addition to UV radiation. The repeated induction of these collagen-degrading enzymes, over the long-term, is thought to cause collagen damage, which is one of the reasons for photoaging. Although several MMPs are expressed in the mammalian skin, it has been suggested that MMP-1 is the major collagen-degrading enzyme responsible for collagen destruction, in severely photo-damaged skin [33]. The upregulation of MMP expression is stimulated by the activator protein-1 (AP-1), which is known to be a UV-inducible transcription factor [34]. In fact, AP-1 regulatory element exists in the 5’ flanking region of MMP genes. Transforming growth factor-beta (TGF-β) and NF-κB are also known to be involved in the induction of MMPs in the skin [34].

### 2.5. Induction of Protein Glycation 

Protein glycation (non-enzymatic glycosylation), also known as the first step of the Maillard reaction, involves the formation of covalent bonds between proteins and reducing sugars. A condensation reaction between the free amino groups of proteins and the carbonyl groups of sugars, results in the formation of a Schiff base, followed by an Amadori product. Excessive products are oxidized and dehydrated to form stable, molecular, cross-linking products, called advanced glycation end-products (AGEs) [35]. Protein glycation influences the physical and functional properties of a protein, as it causes conformational changes in the protein structure [36]. In skin, it has been reported that glycation of collagen type I is associated with the development of skin dullness and decreased skin elasticity [37]. AGEs are also involved in the generation of ROS. Masaki et al. reported that exposure of AGEs to UV-A irradiation in vitro, resulted in the generation of ROS, such as ∙O_2_^−^, H_2_O_2_, and ∙OH, as mentioned above [24]. In humans, skin autofluorescence, a biomarker for AGEs, can function as an endogenous photosensitizer that induces ROS generation, following exposure to UV-A radiation [35]. Thus, a glycation reaction followed by AGE formation is thought to be one of the fundamental mechanisms associated with skin aging, under environmental conditions, especially UV radiation [24].

## 3. Natural Compounds that Prevent UV radiation (UVR)-Induced Photo-Damage

Wavelength dependency is crucial in determining photobiological effects. Short-wavelength UV-C is the most damaging type of UV, but because it is filtered out by the ozone layer, only UV-B and UV-A are considered to be of biological significance. Considering their impacts, only UV-A causes indirect DNA damage, via the generation of ∙O_2_^−^, an initiator of important ROS (see Section 2.2). UV-A penetrates deep into the dermis of the skin. Prolonged UV-A exposure can lead to photoaging and suppression of the immune system. Direct DNA damage, however, is caused by UV-B, via the formation of CPDs and 6-4PPs. UV-B also causes skin damage and transformation, for example, by affecting skin structures and causing wrinkling (a sign of photoaging), roughness, and premature aging, and even leading to skin malignancies and fatal diseases, such as skin cancers [38]. 

To prevent or ameliorate these adverse effects, the blockage of excessive UV or alleviating the cascade of UV-induced photo-damage is crucial. Sunscreen agents (either physical blockers or chemical agents) are often applied to the skin to block excessive UV. Recently, there has been a growing demand for artificial chemical sunscreens to be replaced with natural ones. This is because of the adverse or side effects of artificial chemical sunscreens, and because of environmental concerns. Natural sunscreens and natural compounds that possess properties that resist UV-induced photo-damage are, therefore, of great interest. Some natural compounds can lead to significant improvements in skin health and performance. 

Natural compounds that can provide protection against UVR-induced photo-damage can be categorized on the basis of these mechanisms—(1) blockage of UV photons; (2) involvement in the DNA repair system; (3) antioxidant activity; (4) anti-immunomodulatory activity; (5) anti-inflammatory activity; and (6) having inhibitory action on the cellular matrix [39]. In this section, we describe the natural compounds from plant extracts and a class of secondary metabolites which have pharmacological relevance as superior sunscreen compounds, called mycosporine-like amino acids (MAAs).

### 3.1. Plant Extracts

The database of natural compounds (NCs) contains more than 320,000 compounds, reported in Super Natural II (https://doi.org/10.1093/nar/gku886). Of these, more than 50,000 compounds obtained from plant extracts have been submitted to the database (http://kanaya.aist-nara.ac.jp/KNApSAcK_Family/). The largest class of natural plant extracts which have been shown to have the most relevant activity and the most beneficial effects against UV-B induced photo-damage reported to date, are those which work via inhibitory effects on the signaling pathways (for example, NF-κB, MARK, and AP-1), and biomarker proteins of the target signaling pathways (for example, COX-2 in inflammatory signaling). Inhibitory effects on these signaling pathways further modulate cellular signaling targets and antioxidant properties, to deplete ROS generation. For example, pomegranate fruit extract has been demonstrated to inhibit UV-B-mediated phosphorylation of the NF-κB and MARK pathways, in normal human epidermal keratinocytes (NHEK) [40]. Inhibition of UV-B-induced activation of MARK and NF-κB by proanthocyanidins, a class of polyphenols isolated from grape seeds, polyphenols from green tea, and quercetin from onion, have been reported [41]. Resveratrols, a class of natural phenols obtained from grape skin, cranberries and peanuts, possess the capacity to reduce NF-κB activation [42]. COX-2, a biomarker protein of the inflammatory cascade, is inhibited by phenolic compounds, such as caffeic acid from white grapes [43], kaempferol from grapes [44], and curcumin from turmeric [45]. Comprehensive studies into natural plant agents that can protect against UVB-induced photo-damage have been summarized in previous review papers [46,47,48].

### 3.2. Mycosporine-Like Amino Acids

Mycosporine-like amino acids (MAAs) were first discovered in fungi, associated with a physiological role in light-stimulated sporulation. In 1993, a direct effect as a photo-protectant was made evident by the compound’s ability to block photons. It was revealed that MAAs could prevent 3 out of 10 photons from hitting a cytoplasmic target in cyanobacteria [49]. This ability has led MAAs to be known as a primary sunscreen. 

Accumulating evidence has shown that some MAAs possess additional beneficial functions. Their pronounced photo-protective potential was first noted when they were proved to be ROS scavengers, an ability which arose via their antioxidant ability [14,50]. Further, distinct biological functions have since been proved, including DNA damage-protection, anti-inflammatory activity, and inhibitory action toward AGEs. These multiple beneficial functions make MAAs very interesting biomolecules. More details of these natural compounds will be further reviewed in the next section.

## 4. Molecular Properties of MAAs

In this section, the current basic understanding of MAAs is summarized in three parts—a general description of MAAs, their chemical structure, and their biosynthetic pathways.

### 4.1. General Description

**Figure 3:**
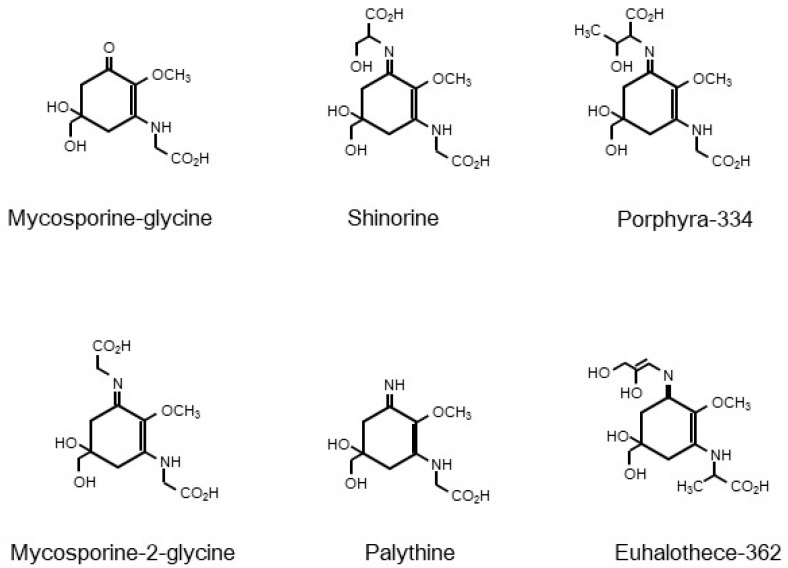
Chemical structures of the selected mycosporine-like amino acids (MAAs)—mycosporine-glycine, shinorine, porphyra-334, mycosporine-2-glycine, palythine, and euhalothece-362.

‘Mycosporine’ is a common term used for fungal UV-absorbing metabolites that have been substituted with amino acid residues [51]. Since the 1960s, mycosporine derivatives, grouped as MAAs, have been found and identified from a wide range of organisms, including marine organisms such as red algae, sea stars, corals, dinoflagellates, cyanobacteria, and lichens [12,52]. MAAs comprise a cyclohexenone or cyclohexenimine ring, as their core chromophore ring structure substituted with amino acid residues or imino alcohols, or some further modifications (Figure 3). For example, in the structure of P334, threonine and glycine bind to the C1 and C3 positions of the core chromophore structure, respectively.

MAAs are considered to be the most effective UV-A-absorbing compounds in nature [53]. MAAs exhibit a maximum absorbance within the UV-A and UV-B range (from 310 to 362 nm), with high molar extinction coefficients (ε = 28,100–50,000 M^−1^cm^−1^). In organisms that accumulate MAAs, these compounds are believed to contribute to the suppression of UV-induced stress, by dissipating excess energy in the form of heat, without generating ROS, after absorbing UV radiation. MAAs are highly water-soluble compounds, due to their zwitterionic properties, derived from their amino acid substitutions, and therefore, they generally accumulate in the cytosolic space. It has been reported that MAAs might act as multifunctional compounds within cells. Besides their UV-protective role, another important biological role of MAAs is their antioxidant properties [14]. Other properties of MAAs, proposed so far, include DNA-protective activity [54], anti-inflammatory activity [55,56], activity to promote osmotic equilibrium [57,58], and involvement in cell–cell interactions [13].

### 4.2. Chemical Structure

MAAs can be roughly divided into two groups—mono-substituted MAAs and di-substituted MAAs. In mono-substituted MAAs, the C3 position on the cyclohexenone structure is substituted with an amino compound. One of the mono-substituted MAAs, mycosporine-glycine (MG), which is a common intermediate for the bioproduction of di-substituted MAAs, has been suggested to be an important protectant against sunlight damage in marine organisms, via the elimination of ^1^O_2_ [59]. The absorption maximum of MG is reported to be 310 nm. On the other hand, the absorption maxima of di-substituted MAAs, vary from 320 to 362 nm, depending on the type of substituent. For example, the absorption maxima of P334 (C1: Thr; C3: Gly), palythine (C1: −NH_2_; C3: Gly), and M2G (C1: Gly; C3: Gly) are 334, 320, and 331 nm, respectively [14]. In di-substituted MAAs, a protonated nitrogen atom on the imine group, results in the formation of a zwitterion, followed by conjugation and delocalization of the positive charge on the nitrogen atom, over the core ring structure. This conjugation stimulates UV absorption by MAAs. The extent of resonance delocalization can affect the extinction coefficient and absorption maximum of each MAA [14]. Further modification of the substituents of MAAs by condensation, dehydration, decarboxylation, oxidation, reduction, sulfonation, or glycosylation might also affect them.

To date, more than 30 different MAAs have been identified. To identify and characterize these MAAs, a variety of experimental techniques have been used, such as high-performance liquid chromatography (HPLC) analysis, mass spectrometry (MS) analysis including liquid chromatography (LC)-MS, amino acid analysis, infrared (IR) spectroscopic analysis, nuclear magnetic resonance (NMR) analysis, and gas chromatography (GC)-MS analysis. An appropriate combination of these analytical techniques, along with technological improvements in analytical instruments, has been effective in helping to characterize the structure of MAAs [12]. To prepare MAA materials for analysis, preparative liquid chromatographic techniques have often been used, following the extraction of MAAs from organisms using organic solvents, such as methanol and ethanol [60]. 

### 4.3. Biosynthetic Pathways

The molecular factors involved in the biosynthetic pathways of MAAs have so far mainly been identified and analyzed in cyanobacteria. Gene clusters for MAA bioproduction in cyanobacteria, generally consist of four genes. For example, MAA synthetic gene clusters in *Anabaena variabilis* ATCC 29413, *Nostoc punctiforme* ATCC 29133, *Aphanothece halophytica*, and *Microcystis aeruginosa* PCC 7806, contain *Ava_3858* to *Ava_3855, NpR5600* to *NpF5597, Ap3858* to *Ap3855,* and *mysA* to *mysD*, respectively [13,61,62]. It has been reported that *A. variabilis* ATCC 29413, *N. punctiforme* ATCC 29133, and *M. aeruginosa* PCC 7806, produce mainly SHI, whereas M2G is known as the sole MAA produced by *A. halophytica*. The first two genes encode predicted DHQ synthase (DHQS) and O-methyltransferase (O-MT), respectively. The protein products of these two genes are predicted to synthesize 4-deoxygadusol (4-DG), a common precursor compound for the synthesis of MAAs, from 3-dehydroquinate (DHQ), a shikimate-pathway intermediate, or sedoheptulose-7-phosphate (SHP), an intermediate of the pentose phosphate pathway [10,61]. The protein products of the third genes, which encode the adenosine triphosphate (ATP)-grasp enzyme superfamily, catalyze the imine linkage of 4-DG, with glycine, to produce MG [61]. Finally, the protein products of the fourth genes, which encode a non-ribosomal peptide synthase (NRPS)-like protein or D-Ala-D-Ala ligase, yield di-substituted MAAs, from MG, by the attachment of an additional amino acid moiety. This last step for di-substituted MAA-bioproduction by the fourth protein, might cause the attachment of different amino acid residues, due to differences in substrate specificity. In addition to cyanobacteria (although there is still room for further functional molecular characterization), homologues of these cyanobacterial MAA synthetic genes have been found in fungi, actinobacteria, dinoflagellates, sea anemones, and corals [61,63,64].

Although detailed investigations of the regulatory molecular mechanisms for MAA bioproduction remain to be done, a number of studies have revealed that various environmental factors might affect intracellular MAA accumulation levels in MAA-producing organisms. UV exposure is known to be a typical MAA production-enhancing factor [10]. In addition to UV irradiation, other abiotic stresses, such as salt stress, thermal stress, and nutrient availability, can induce MAA production [12]. For example, salt stress upregulated the expression of M2G synthetic genes and increased M2G production in *A. halophytica*, and a combination of UV-B irradiation and salt stress, resulted in the enhancement of M2G accumulation, compared with UV-B exposure only [62]. In this cyanobacterium, the oversupply of nitrate, glycine, or serine, also induced intracellular M2G accumulation [65]. Enhanced MAA production has been observed in some corals, when simultaneously exposed to UV and thermal stresses, whereas exposure to high solar radiation, which contains both photosynthetically active radiation (PAR) and UV radiation, and thermal stress, was seen to decrease MAA production in the Caribbean coral *Montastraea faveolata* [10]. Thus, it has been suggested that there is complex regulation of MAA bioproduction, in response to these environmental stresses.

## 5. Potential of Anti-Photoaging and Photo-Protective Activity of MAAs

In this section, experimental observations for understanding the potential of MAAs, in terms of their anti-photoaging and photo-protective activities, are summarized in six parts—DNA damage-protecting activity, anti-oxidative activity, anti-inflammatory activity, anti-protein glycation activity, collagenase inhibitory activity, and other activities.

### 5.1. DNA Damage-Protecting Activity 

DNA damage can be caused both directly by UV-B and indirectly by UV-A, via the formation of ROS. It has been reported that MAAs have the potential to protect DNA against damage from oxidative stress induced by the ROS, H_2_O_2_ [54]. This study was performed using the A375 human melanoma cell line, a model used for the study of the development of skin cancer. M2G rescued DNA from the damage induced by H_2_O_2_. Using the comet assay, it was demonstrated that M2G had a somewhat high genoprotective effect, similar to ascorbic acid. This direct in vivo assay, thus, revealed the potential role of M2G in protecting against DNA damage caused by oxidative stress induced by H_2_O_2_.

The efficacy of protecting against DNA damage by another well-known MAA, P334 (in the form of Helioguard^®^365), originally isolated from red algae, was evaluated. In this case, the fibroblast cell line IMR-90 was used as the model in the study [66]. Protective activity against DNA damage caused by UV-A was observed in the presence of Helioguard^®^365. A visible reduction in DNA damage in the presence of Helioguard^®^365 occurred in a dose-dependent manner. Palythine also exhibited a protective effect against UV-A-induced DNA damage in HaCaT cells [67]. This is a cell line of immortalized human keratinocytes, which has been extensively used to study epidermal homeostasis. The formation of cyclobutane pyrimidine dimers (CPD), 8-oxo-7,8-dihydroguanine (8-oxoGua), and alkali-labile sites (ALS), was drastically reduced in palythine-treated HaCaT cells subjected to UV-A irradiation. Results obtained either from purified MAAs (such as M2G and palythine) and MAAs in formulation (Helioguard^®^365), indicated the preventive efficacy of MAAs, against DNA damage caused by direct oxidative stress, due to ROS.

### 5.2. Antioxidant Activity

In biological processes, oxidation is essential for energy metabolism and production. It has long been recognized that energy metabolism is linked to ROS production. The ROS produced can serve as cell signaling molecules, triggering cellular processes, such as cell division, inflammation, immune functions, and stress responses [68]. These molecular and cellular mechanisms are under the tight control of the equilibrium of ROS generation and scavenging. During exposure to UV, or following some oxidative reactions (such as contact with foreign chemicals), ROS can be constantly generated. ROS generation mediated by UV radiation has been shown to stimulate the expression of genes in signaling pathways, which can consequently exert several physiological effects, including inflammation and protein oxidation. To suppress photo-oxidation or scavenging ROS, antioxidant defense mechanisms are vital.

Substantial evidence has revealed that MAAs have potential abilities as antioxidants [14,52,54,60,67,69,70]. Various MAAs have the ability to scavenge such ROS, like hydroxyl radicals, hydroperoxyl radicals, singlet oxygen, and superoxide anions. The antioxidant role of MAAs might have a special significance in scavenging the free radicals propagated by oxidative stress induced by UV radiation or other environmental stresses. Among the 30 MAAs currently known, antioxidant activity has been clearly observed in some of mono-, di-substituted MAAs, and glycosylated MAAs, in both, in vitro and in vivo studies [14,52,54,60,67,69,70]. It should be noted that some MAAs showed indirect evidence for antioxidant capacity, via a slow photodegradation in the presence of a photosynthesizer, or a reaction with singlet oxygen [14]. These observations will not be included in this review. Table 1 summarizes radical scavenging activity of MAAs. IC50 values against organic radical sources, such as DPPH (2,2-diphenyl-1-picryhydrazyl), ABTS (2,2’-azino-bis(3-ethylbenzthiazoline-6-sulphonic acid), and an oxygen radical absorption capacity (ORAC) were reported.

As summarized in Table 1, various research groups performed in vitro analyses to assess antioxidant activity, for example using an organic radical, such as a DPPH assay; an organic cation radical, such as an ABTS assay; and an ORAC assay. The DPPH assay revealed that M2G exhibited the strongest antioxidant properties, followed by MG, while SHI and P334 showed these properties to a lesser extent [54,60,71]. The ABTS assay showed a similar trend to the DPPH assay, with M2G exhibiting the strongest oxygen-radical absorption capacity, followed by SHI and P334 [60]. Glycosylated MAAs seem to display slightly different antioxidant capacities. In some cases, a slow-acting radical scavenging was observed. Among the three glycosylated MAAs, the hexose-bound-P334 [72], 7-*O*-(β-arabinopyranosyl)-P334 extracted from the cyanobacterium *Nostoc commune* [72,73], and 13-*O*-β-galactosyl-P334 from *Nostoc sphaericum* [69], the 7-*O*-(β-arabinopyranosyl)-P334 showed the highest activity. These glycosylated MAAs were derived from P334, and the scavenging activity observed suggested that their glycosylation led to an increase in scavenging activity. Variations in the MAA chemical structure is one of the most interesting features of this class of compounds, particularly with regard to the structure–activity relationships. Structure–activity relationships of the biologically active MAAs have been discussed elsewhere [14,71]. However, the exact mechanisms of these biologically active MAAs are still unknown, and detailed investigations will be a useful direction for future studies. In addition to MAAs, other compounds exhibiting antioxidant activity, such as scytonemins, phenols, isobenzofuranone derivatives, exopolysaccharides, diketopiperazine alkaloids, and dioxopiperazine alkaloids, have been found in various organisms, including marine fungi and bacteria [16]. For the therapeutic application purpose, taking into account that naturally occurring products have been proven to be relatively safer, it could be preferred to utilize these natural antioxidants for human utilization, instead of synthetic ones. Among these natural compounds, scytonemins are a class of well-known natural cyanobacterial UV sunscreen, with basic characteristics such as hydrophobic pigments. The antioxidant property of scytonemins was revealed by an electron spin resonance analysis and ABTS assay. For example, the purified scytonemin from *Nostoc commune* displayed a radical scavenging activity, with an IC50 value of 36 µM, which was comparable with MAAs [11]. Carotenoids, the most common pigments in nature, are also known for their antioxidant activity, as well as light harvesting and photoprotective functions in photosynthetic organisms [11]. Among over 750 carotenoids found so far, astaxanthin is believed to be one of the strongest antioxidants in nature [74]. Recently, Dose et al. estimated the 50% scavenging concentration (SC50) value of astaxanthin, for the DPPH free radical scavenging, to be around 500 μM [75]. On the other hand, Cheewinthamrongrod et al. determined SC50 values of M2G and MG, for DPPH free radical scavenging, as 22 μM and 43 μM, respectively [54]. Although the assays for measurement of DPPH free radical scavenging activities were not identical (electronspin resonance spectroscopy (ESR) method combined with spin trapping for astaxanthin; colorimetric method for M2G and MG), these observations suggest that a certain kind of MAAs are powerful natural antioxidant molecules, derived from marine organisms. It would be desirable that further comprehensive research analyses elucidate the significance of MAAs as antioxidative molecules, by comparing other natural compounds. Apart from the antioxidant capacity obtained from in vitro and in vivo studies, experimental evidence suggests certain MAA, such as M2G and palythine, have the necessary characteristics of biocompatible natural compounds, to protect the human skin [54,67]. M2G is biocompatible with normal human skin fibroblast cells [54]. Palythine significantly reduced a wide range of adverse effects from UV-radiation-induced damage in HaCaT keratinocytes. The combined experimental evidence, either from antioxidant capacity, biocompatibility, or several other lines of evidence, suggest that MAAs have a superior function, compared with other antioxidants. 

Oxidative stress can trigger several signaling pathways, and of these, Kelch-like ECH-associated protein 1/nuclear factor erythroid 2-related factor 2/antioxidant response element (Keap1/Nrf2/ARE) signaling, was shown to be the major mechanism in alleviating oxidative stress in human cells, via the regulation of antioxidant and detoxification enzymes [68]. A recent study found that M2G suppressed the expression of the transcription factor Nrf and the antioxidant-associated genes encoding the detoxification enzymes Cu/Zn-superoxide dismutase (*Sod1*), catalase (*Cat*), and heme oxygenase-1 (*Hmox1*) in RAW 264.7 macrophage cells, under oxidative stress induced by H_2_O_2_. The enzymatic activities of SOD and CAT were also found to be attenuated, in agreement with the transcriptional analysis.

In Keap1/Nrf2/ARE signaling, the transcription factor Nrf2 is generally attached to Keap1, forming a Keap1/Nrf2 complex. This inactivated protein is retained in the cytosol, by the binding of Keap1 with actin or myosin. Activation of the Keap1/Nrf2/ARE pathway only occurs after the detachment of Keap1 and Nrf2. This step is induced by oxidative species and electrophiles. Activated Nrf2 is then localized in the nucleus, where it binds to the basic leucine zipper-musculoaponeurotic fibrosarcoma (bZip-Maf) protein, at the ARE region. Finally, the interaction between heterodimers and the ARE-promotor region, initiates the transcription of antioxidative genes [76,77,78]. It has been demonstrated that SHI and P334 have the ability to bind with Keap1, associated protein in Keap1/Nrf2/ARE signaling [70]. The ability of MAAs to dissociate Nrf2 from Keap1, was confirmed by the up-regulation of mRNA expression of the Nrf2-targeted genes, which encode oxidative-stress defense proteins in primary skin fibroblasts, prior and post UVR exposure. This molecular evidence suggests that SHI and P334 are activators of the Keap1/Nrf2 signaling pathway, and thus, have beneficial effects for antioxidative gene expression.

### 5.3. Anti-Inflammatory Activity

Inflammation is a vital component of the physiological defense process, in response to molecular and cellular damage caused by oxidative stress, irradiation, infection, and exposure to endotoxins, such as lipopolysaccharides (LPS) [79]. Oxidative stress can directly induce inflammation through the canonical pathway [80]. Conversely, UV radiation activates the trigger protein elF2α, inducing expression of GCN/PERK2 [81].

To date, the anti-inflammatory activities of MAAs have been investigated in human keratinocyte, HaCaT, and RAW 264.7 macrophage models. Suh et al. [56] evaluated the effects of SHI, P334, and MG, on the expression of genes associated with inflammation, using the human fibroblast cell line, HaCaT, in response to UV irradiation. Among these MAAs, only MG suppressed the expression of an inflammation marker gene, COX-2, and in a concentration-dependent manner. An in vitro model comprising RAW 264.7 macrophages was used to evaluate anti-inflammatory effects, in response to stimulation by LPS. Nitric oxide (NO) is an important pro-inflammatory signaling molecule, and is considered to be a good index of inflammation [82,83]. It was shown that M2G exhibited the most potency in reducing NO production, in response to LPS inflammatory stimulation, with effects that were two- to three-fold higher, compared with SHI, P334, and palythine [71]. Another line of anti-inflammatory effects became evident, following the transcriptional analysis. M2G-pre-treatment of RAW 264.7 cells, stimulated by LPS, significantly suppressed the expression of the key inflammatory signaling regulatory genes iNOS and COX-2. The up-regulation of iNOS and COX-2, during inflammation, is controlled by the pro-inflammatory transcription factor NF-κB, therefore, it seems likely that M2G inhibits the production of inflammatory mediators, by suppressing the NF-κB pathway. M2G inhibition of iNOS and COX-2 expression in activated macrophages, is regarded as a potentially interesting tool for the treatment or prevention of inflammation.

### 5.4. Anti-Protein-Glycation Activity

Glycation of proteins leads to the generation of AGEs, which are linked to the progression of aging and age-related diseases. It was recently reported that MAAs can have inhibitory effects on protein glycation [84]. In that report, the glycation-dependent cross-linking of hen egg white lysozyme (HEWL), which is a structural homologue of human lysozyme, was evaluated with or without addition of M2G, or a mixture of P334 and SHI. Both samples with added MAAs showed inhibitory activity, with M2G isolated from *A. halophytica* showing a greater activity than the mixture of P334 and SHI. The 50% inhibitory concentration (IC50) value for the HEWL dimer formation with M2G, was 1.61 mM. This value was less than that of the aminoguanidine, which is known to be an inhibitor of glycation via reactions with the Amadori carbonyl groups of glycated proteins. These observations suggest that MAAs, and M2G, in particular, can be useful in preventing the formation of AGEs. Further studies using other proteins in addition to HEWL, such as collagen type I, which is known to be associated with skin-aging, will be an interesting avenue for future research. Although detailed investigations are needed to fully understand the glycation-inhibitory activity of MAAs, the antioxidant properties of MAAs, might contribute to this activity, since some antioxidants, including aminoguanidine, inhibit protein glycation, by preventing oxidation of the Amadori product [85]. Taking into account the greater antioxidant activity of M2G, compared with a mixture of P334 and SHI (see above), this hypothesis is reasonable.

### 5.5. Bacterial Collagenase Inhibitory Activity

Mammalian collagenases that belong to the matrix metalloproteinase (MMP) family are important enzymes for the maintenance of skin homeostasis, through the destruction of ECM proteins; they are involved in the skin-aging process, as described above. On the other hand, bacterial collagenase, which is one of the factors of bacterial virulence, enables the destruction of the extracellular structure, by attacking the collagen helix, and is responsible for part of the pathogenic process in some bacteria, such as *Clostridium* [86]. Thus far, two research groups have reported that MAAs might inhibit bacterial collagenase activity. One group found that SHI, P334, and palythine inhibited *Clostridium histolyticum* collagenase activity [87]. The IC50 values were 104.0, 105.9, and 158.9 μM for SHI, P334, and palythine, respectively. On the other hand, another group showed that both M2G and a mixture of P334 and SHI, inhibited the *C. histolyticum* collagenase activity with IC50 values of 0.47 and more than 10 mM, respectively, in the presence of calcium chloride [84]. Even though the range of IC50 values between these two reports is quite wide, probably due to differences in the assay procedures, these observations indicate that M2G possesses the greatest inhibitory activity among the MAAs tested so far. The inhibitory mechanism of MAAs remains unknown. However, the metal chelating activity of MAAs might play a role in this property because collagenases are metalloproteases. Tarasuntisuk et al. reported that M2G showed a metal chelating activity, when using iron (II) chloride, whereas a mixture of P334 and SHI did not show any remarkable activity [84]. Therefore, the strong inhibitory effect of M2G might be due to the chelation of calcium ions in the reaction system. Besides M2G, euhalothece-362 from the cyanobacterium *Euhalothece* sp. strain LK-1, has also been suggested to be an MAA that acts as an iron chelator [88]. In addition to their effect on bacterial collagenase, the effect of MAAs on mammalian collagenases is also an interesting subject that requires further investigation.

Another protease involved in ECM-degradation is elastase, which is a member of the chymotrypsin-type serine protease family [89]. Elastase can break down an important protein, elastin, within the ECM, and in the absence of metal ions. Degradation of elastin reduces skin elasticity. Our preliminary investigation into elastase from porcine pancreas showed that the purified MAAs tested exhibited no inhibitory activities; M2G, P334, and SHI, were at final concentrations of 4.0, 7.0, and 5.3 mM, respectively. This result again suggests a link between the metal chelating activity and collagenase inhibitory activity in MAAs.

### 5.6. Other Activity

In addition to the aforementioned anti-photoaging and photo-protective activity of MAAs, in this section we describe our unpublished observations following our testing of the tyrosinase inhibitory activity of MAAs. Melanin, a key pigment that plays an important role in protecting the skin against UV damage, is associated with abnormal pigmentation and melanoma. Overaccumulation of melanin can induce certain types of skin disorder [90]. Tyrosinase is known to be an enzyme involved in melanin biosynthesis. To explore whether tyrosinase activity could be inhibited, the effects of purified M2G, SHI, and P334 were tested with mushroom tyrosinase. None of the MAAs tested affected the tyrosinase activity, at maximum final concentrations of 6.4, 8.4, and 7.5 mM M2G, SHI, and P334, respectively; whereas, 60 μM of a standard inhibitor, kojic acid, inhibited activity by more than 50%. Thus, although there may still be scope for further investigations, the MAAs tested did not show any inhibitory activity toward tyrosinase. As with the research described here, other anti-aging-related activities of MAAs remain to be clarified. For example, an investigation into the possible inhibitory effects of MAAs on hyaluronidase, which can contribute to collagen breakdown in the skin, would be another interesting anti-aging feature of MAAs to characterize. Observations relating to these properties, including negative results, will be important for the future development and application of MAAs as potential therapeutic agents.

## 6. Concluding Remarks

One of the greatest risk factors for skin-aging is UV radiation. In this review, the protective properties of MAAs were discussed, alongside the challenges for prevention of UV-induced skin-aging. Of the MAAs that were discussed, we highlighted the ability of M2G in particular. M2G exhibits prominent abilities for protecting DNA against UV-related damage, as well as antioxidant, anti-inflammatory, anti-protein-glycation, and collagenase inhibition activities. These observations indicate the potential of M2G for therapeutic applications. However, many promising MAAs, including MAA derivatives, such as glycosylated MAAs, still need to be studied in detail, since the variety of MAAs examined to date is not adequate. In the future, a full understanding of the relationship between the chemical structure of MAAs and their activity, might help to achieve the development and commercialization of MAAs, for multipurpose uses in the cosmeceutical, pharmaceutical, biomedical, and biotechnological fields. In this regard, marine organisms, including cyanobacteria, and green and red macroalgae, are promising candidates as environment-friendly sources of industrially important compounds, like MAAs, because of their photoautotrophic properties, which can convert solar energy and carbon dioxide into useful chemicals.

## Figures and Tables

**Table 1:** IC50 values against 2,2-diphenyl-1-picryhydrazyl (DPPH), 2,2’-azino-bis(3-ethylbenzthiazoline-6-sulphonic acid (ABTS), and oxygen radical absorption capacity (ORAC) of mono-, di-substituted MAAs, and glycosylated MAAs.

Mycosporine-like Amino Acids (MAAs)	IC50	References
*Mono-Substituted MAAs*		
Mycosporine-glycine	3 µM ^a^ at pH 8.5	[14,52]
	43 µM ^b^	[54]
Mycosporine-γ-aminobutyric acid	0.6 mM ^a^	[14]
*Di-Substituted MAAs*		
Mycosporine-2-glycine	45 µM ^a^	[54]
	22 µM ^b^	[71]
Palythine	21.3 µM ^b^	[67]
	714 µM ^c^	[67]
Porphyra-334	133 µM ^a^	[60]
	185.2 µM ^b^	[70]
Shinorine	94 µM ^a^	[60]
	399 µM ^b^	[70]
*Glycosylated MAAs*		
Hexose-bound-P334	58 mM ^a^	[69]
7-*O*-(β-arabinopyranosyl)-P334	9.5 mM ^a^	[69]
13-*O*-β-galactosyl-porphyra-334	17 mM ^a^	[69]
*Standard antioxidants*		
Trolox	10 µM ^b^	[60]
Ascorbic acid	21.3 µM ^b^	[67]
α-Tocopherol	11.1 µM ^b^	[67]

^a^ Radical scavenging activity measured using ABTS as he organic radical source. ^b^ Radical scavenging activity measured using DPPH as the organic radical source. ^c^ Radical scavenging activity measured using the ORAC antioxidant assay kit.
